# The synergistic effect of *Levilactobacillus brevis* IBRC-M10790 and vitamin D3 on *Helicobacter pylori*-induced inflammation

**DOI:** 10.3389/fcimb.2023.1171469

**Published:** 2023-05-05

**Authors:** Ali Nabavi-Rad, Shaghayegh Jamshidizadeh, Mahsa Azizi, Abbas Yadegar, Karen Robinson, Tanya M. Monaghan, Mohammad Reza Zali

**Affiliations:** ^1^ Foodborne and Waterborne Diseases Research Center, Research Institute for Gastroenterology and Liver Diseases, Shahid Beheshti University of Medical Sciences, Tehran, Iran; ^2^ Basic and Molecular Epidemiology of Gastrointestinal Disorders Research Center, Research Institute for Gastroenterology and Liver Diseases, Shahid Beheshti University of Medical Sciences, Tehran, Iran; ^3^ National Institute for Health Research Nottingham Biomedical Research Centre, University of Nottingham, Nottingham, United Kingdom; ^4^ Nottingham Digestive Diseases Centre, School of Medicine, University of Nottingham, Nottingham, United Kingdom; ^5^ Gastroenterology and Liver Diseases Research Center, Research Institute for Gastroenterology and Liver Diseases, Shahid Beheshti University of Medical Sciences, Tehran, Iran

**Keywords:** *Helicobacter pylori*, *Levilactobacillus brevis*, vitamin D3, extracellular vesicles, synergistic effect, AGS cells

## Abstract

**Background:**

Owing to the emergence and spread of multidrug resistance mechanisms in *Helicobacter pylori*, achieving a successful eradication has become exceedingly difficult. Thus, this study for the first time determines the effect of a combination of vitamin D3 and probiotic on the pathogenesis and treatment of *H. pylori*.

**Methods:**

We established an *in vitro* experimental system using AGS human gastric carcinoma cells and explored the synergistic effect of *Levilactobacillus brevis* IBRC-M10790 and vitamin D3 on *H. pylori*. Live and pasteurized *L. brevis*, *L. brevis*-derived membrane vesicles (MVs), and *L. brevis* cell-free supernatant (CFS), as well as their combination with vitamin D3 were used during this study. We assessed the anti-inflammatory and anti-oxidative effects of these combinations using RT-qPCR and ELISA, respectively. We further performed an adhesion assay to evaluate the influence of *L. brevis* and vitamin D3 on the adherence rate of *H. pylori* to AGS cells.

**Results:**

Our results demonstrated that *L. brevis* and vitamin D3 possess anti-inflammatory and anti-oxidative effects against *H. pylori* infection in AGS cells. The combination of vitamin D3 with the probiotic strain (particularly live *L. brevis* and its CFS) can more efficiently reduce the expression of pro-inflammatory cytokines IL-6, IL-8, IFN-γ, and TNF-α in the AGS cells. Moreover, vitamin D3 and *L. brevis* exhibited an additive impact preserving the integrity of the epithelial barrier by increasing the expression of the tight junction protein ZO-1. Furthermore, this combination can potentially reduce *H. pylori* adherence to AGS cells.

**Conclusions:**

This study indicates the advantage of combining vitamin D3 and probiotic to attenuate *H. pylori*-induced inflammation and oxidative stress. Consequently, probiotic and vitamin D3 co-supplementation can be considered as a novel therapeutic approach to manage and prevent *H. pylori* infection.

## Introduction

Infection with *Helicobacter pylori* (*H. pylori*) is the primary causative factor in the development of chronic gastritis, peptic ulcer, and gastric carcinoma (Fakharian et al., 2022). *H. pylori* might be the most successful of human pathogens, considering the global colonization prevalence of almost 50%. Owing to the profound influence of socioeconomic factors on the prevalence of *H. pylori* infection, its colonization rates exceeds 80% in parts of Eastern Asia and European countries (O’Connor et al., 2017). This in principle has resulted in the recommendation that all *H. pylori*-infected patients should receive treatment. Therefore, all international consensus conferences have advocated a cost-effective *H. pylori* test-and-treat strategy for dyspeptic patients, which is mainly based on a non-invasive *H. pylori* diagnostic test and subsequent eradication when detected (Beresniak et al., 2020). The standard *H. pylori* eradication regimen consists of two or three types of antibiotics (mostly clarithromycin, amoxicillin, and metronidazole) and a proton-pump inhibitor (PPI) for two weeks (Suzuki et al., 2020).

The test-and-treat strategy has become challenging owing to the increasing rate of single-drug and multi-drug resistance of *H. pylori* to previously effective antibiotic-based treatments (Malfertheiner et al., 2022). Consequently, the interest in unconventional therapeutic strategies is growing by the day. In the last decade, probiotic co-supplementation to conventional antibiotic therapies has been demonstrated to attenuate the deleterious effects of antibiotics, modulate the host immune response, and orchestrate an anti-inflammatory response. The regular uptake of probiotics has been suggested to reduce the available bacterial attachment sites, therefore reducing *H. pylori* adhesion to gastric epithelial cells (Nabavi-Rad et al., 2022b). Consequently, probiotic administration, especially *Lactobacillus* spp., is currently considered to be an effective concomitant treatment strategy for *H. pylori* infection (Ji and Yang, 2020). The expanding supplementation of probiotics to vulnerable population require concise and actionable guidelines on how to prevent long-term risks and adverse events, such as disrupting the inherent structure of the gut microbiome and transferring antibiotic resistance genes and toxins (Merenstein et al., 2023). While the administration of live biotherapeutics may give rise to safety concerns, postbiotics which are preparations of inanimate microorganisms and/or their components, can confer selective health benefits, and may be safer and more stable alternatives to probiotics (Hitch et al., 2022; Mosca et al., 2022).

Micronutrient malabsorption or deficiency is a well-known characteristic of *H. pylori* infection (Nabavi-Rad et al., 2022a). Recent studies have shed light on the inverse correlation between the serum levels of certain micronutrients, particularly vitamin D, and the risk of *H. pylori* infection and eradication failure (El Shahawy et al., 2018; Han et al., 2019). A number of studies further investigated the beneficial effect of vitamin D supplementation and demonstrated its potential modulatory activity on *H. pylori* pathogenicity (Yang et al., 2019). Owing to its high stability, cholecalciferol (vitamin D3) is the primary supplementary form of vitamin D that more efficiently increases the serum levels of vitamin D, compared to ergocalciferol (vitamin D2) (Vieth, 2020).

Evidence of synergistic health benefits of vitamin D and probiotic co-supplementation is emerging. A systematic review of randomized controlled trials has shown that co-supplementation of vitamin D with probiotic strains of *Lactobacillus*, *Bifidobacterium*, and *Streptococcus* yielded greater health benefits than its comparators, which were placebo, vitamin D, lower vitamin D dose, and probiotics and lower vitamin D dose (Abboud et al., 2020). Considering the tight interaction of *H. pylori* with the gastric concentration of vitamin D and the presence of probiotic strains among indigenous gastric microbiota, we have evaluated for the first time, the synergistic effects of vitamin D3 and the probiotic strain *Levilactobacillus brevis* IBRC-M10790 on the inflammatory and oxidative activity of *H. pylori in vitro*. To this end, we prepared and utilized live *L. brevis*, pasteurized *L. brevis*, *L. brevis*-derived membrane vesicles (MVs), and *L. brevis* cell-free supernatant (CFS) in this study.

## Materials and methods

### Bacterial strains and culture

A *H. pylori* clinical strain BY-1 was obtained from the *H. pylori* biobank of Helicobacter Research Laboratory in the Research Institute for Gastroenterology and Liver Diseases, Shahid Beheshti University of Medical Sciences, Tehran, Iran. The isolate was cultured on Brucella agar plates containing 5% sheep blood for 2-3 days under microaerophilic conditions (5% O_2_, 10% CO_2_, 85% N_2_) at 37°C.

*L. brevis* strain IBRC-M10790 was kindly provided by Takgene Zist Company (Tehran, Iran). The probiotic strain was grown on MRS (De Man-Rogosa-Sharpe) agar (Merck, Darmstadt, Germany) for 48-72 h at 37°C under anaerobic conditions (85% N_2_, 10% CO_2_ and 5% H_2_) created by Anoxomat^®^ Gas Exchange System (Mart Microbiology BV, Holland).

### Pasteurization of *L. brevis*

The MRS broth culture of *L. brevis* yielded an optical density at a wavelength of 600nm (OD600) of 1.00 ± 0.03, equivalent to 10^9^ colony-forming units (CFU) per ml, following 72 h incubation under anaerobic conditions. The bacterial culture was then heat treated (pasteurized) at 70°C for 30 min and immediately stored at -80°C for at least one hour before use.

### Preparation of *L. brevis* CFS

The MRS broth culture of *L. brevis* was adjusted after 48 h to an optical density OD600 of 1.00 ± 0.03, corresponding to 10^9^ CFU/ml. Following incubation, bacterial cells were eliminated by centrifugation at 18,928×g for 15 min and the supernatant was harvested. The obtained supernatant was then filtered through 0.22 μm filters to remove any remaining bacterial cells. *L. brevis* CFS aliquots were stored at -80°C until further use.

### Isolation of MVs from *L. brevis*

The isolation of *L. brevis* MVs was carried out by culturing a loopful of grown colonies on MRS agar in BHI (brain heart infusion) broth supplemented with 0.5% yeast extract, 0.05% (w/v) L-cysteine (Sigma Aldrich, St. Louis, MO, USA), 5 µg/ml hemin, 1 µg/ml vitamin K1 for 24 h under anaerobic conditions. The turbidity of grown cultures was measured with a spectrophotometer and the bacterial concentration was calculated by optical density conversion factor OD600 of 0.5, corresponding to 1.5×10^8^ CFU/ml. A bacterial suspension of 1000 ml was centrifuged at 10000×g for 20 min at 4°C. The resulting supernatant was filtered through 0.22 μm filters to eliminate any remaining residual bacteria. The MVs were acquired by ultracentrifugation of 36 ml of the samples at 150,000×g for 5 h at 4°C. The obtained pellet was washed in PBS (phosphate-buffered saline, pH=7), ultracentrifuged, resuspended in 300 μl of PBS, and stored at -80°C. Prior to freezing, the protein content of the purified MVs was measured using the BCA (bicinchoninic acid) method (DNAbiotech, Tehran, Iran). The absence of LPS (lipopolysaccharide) in the isolated MVs was confirmed using LAL Chromogenic Endotoxin Quantitation Kit (Thermo Fisher Scientific, MA, USA). The protein pattern of MVs was assessed by SDS-PAGE (sodium dodecyl-sulfate polyacrylamide gel electrophoresis).

### MVs characterization

#### Transmission electron microscopy

The isolated MV samples were applied to a 400-mesh copper grid carbon-coated formvar film and stained with 2% uranyl acetate. The structure of samples was examined in a Philips EM208 TEM system with an accelerating voltage up to 100 Kv and the image magnification of 89 kX.

#### DLS

The purified MVs were analyzed by DLS (dynamic light scattering) in a particle size analyzer (Nano-ZS, ZEN3600, Malvern Instruments, UK) to determine the size distribution of the MVs (Vargoorani et al., 2020).

#### Cell culture conditions

The AGS human gastric adenocarcinoma cell line was obtained from the Iranian Biological Resource Center (accession cell no. C10071). The AGS cells were maintained in media containing RPMI-1640 supplemented with 10% FBS (Gibco-Invitrogen, Carlsbad, CA), 1% penicillin–streptomycin and 2 mM l-glutamine under humidified atmosphere with 5% CO_2_ at 37°C. Sub-culturing of cells was carried out by trypsinization when cells reached 80% confluency.

#### Cell viability assay

AGS cells were seeded in 96-well plates at a density of 1×10^5^ cells/well and reached 80% confluent monolayers. Thereafter, cells were treated with the *H. pylori* isolate at a multiplicity of infection of 100 bacteria per cell (MOI 100), live *L. brevis* (MOI 10, 50, and 100), *L. brevis*-derived MVs (1, 10, 50, and 100 μg/ml), *L. brevis* CFS (20% (v/v), pasteurized *L. brevis* (10^9^ CFU/ml), and vitamin D3 (20, 50, 100, 150 nmol) for 24 h. AGS cells were consequently incubated with MTT solution (Sigma Aldrich, St. Louis, MO, USA) at concentration of 5 mg/L for 4 h and then dissolved in 100 μL of DMSO. The cell viability was measured with an absorbance at 570 nm and a reference wavelength of 630 using a microplate reader (Eon, BioTek Instruments, USA). Vitamin D3 treatments were prepared from cholecalciferol dissolved in cell culture grade DMSO (dimethyl sulfoxide). In addition, DMSO solvent control was included in each experiment.

#### Infection and treatment of AGS cells

Exponentially growing AGS control and experimental cultures were seeded at a density of 1×10^6^ cells/well and incubated for 48 h at 37°C. Firstly, AGS cells were infected with the *H. pylori* isolate or treated with vitamin D3, live *L. brevis*, pasteurized *L. brevis*, *L. brevis*-derived MVs, or *L. brevis* CFS for 24 h. Secondly, based on the cell viability results, AGS cells were treated with *H. pylori* and simultaneously with one of the followings: vitamin D3, live *L. brevis*, pasteurized *L. brevis*, *L. brevis*-derived MVs, or *L. brevis* CFS for 24 h. Finally, other wells of AGS cells were simultaneously treated with *H. pylori*, vitamin D3, and one of the followings: live *L. brevis*, pasteurized *L. brevis*, *L. brevis*-derived MVs, or *L. brevis* CFS for 24 h.

#### Adhesion assay

AGS cells were seeded in 96-well plates at a density of 1×10^5^ cells/well and incubated to reach 80% confluent monolayers. Cells were infected with the *H. pylori* isolate and treated with *L. brevis* with or without vitamin D3 for 2 h. The AGS cell monolayer was washed three times with PBS (pH=7) to eliminate the unattached bacteria. To evaluate the number of adhered bacteria, AGS cells were treated with 0.1% saponin for 5 min at room temperature and consequently spread on Brucella agar supplemented with 5% sheep blood. The bacterial CFU and percentage of adhesion was calculated after 3 days of incubation.

#### Oxidative stress assay

Oxidative stress was evaluated in the supernatant of AGS cells using NO (nitric oxide) assay kit (KPG, Tehran, Iran) and MDA (lipid peroxidation) assay kit (KPG, Tehran, Iran). All experiments were performed in triplicate.

#### Total RNA extraction and RT-qPCR

According to the manufacture’s instruction, total RNA was extracted from treated AGS cells using RNeasy Plus Mini Kit (Qiagen, GmbH, Germany) and the acquired RNA samples were assessed by ultraviolet spectroscopy (NanoDrop spectrophotometer, ND-1000, Thermo Scientific, MA, USA). cDNA was synthesized using a BioFACT™ RT-Kit (BIOFACT CO., Ltd. Daejeon, South Korea). The RT-qPCR was performed by Rotor-Gene^®^ Q (Qiagen, GmbH, Germany) real-time PCR system using BioFACT™ 2X Real-Time PCR Master Mix (BIOFACT CO., Ltd. Daejeon, South Korea). The oligonucleotide sequence of primers used for RT-qPCR are listed in **Table 1**.

**Table 1:** Specific primers used for RT-qPCR.

Target gene	Primer sequence (5′ to 3′)	Size of amplicon (bp)	Reference
TNF-α	F: CCCAGGGACCTCTCTCTAATC R: ATGGGCTACAGGCTTGTCACT	84	(Shan et al., 2017)
IL-6	F: GCACTGGCAGAAAACAACCT R: TCAAACTCCAAAAGACCAGTGA	119	(Shan et al., 2017)
IL-8	F: CTCTTGGCAGCCTTCCTGATT R: ACTCTCAATCACTCTCAGTTCT	147	(Shan et al., 2017)
IFN-γ	F: TGGAGACCATCAAGGAAGAC R: GCGTTGGACATTCAAGTCAG	113	(Caruso et al., 2014)
ZO-1	F: CGGTCCTCTGAGCCTGTAAG R: GGATCTACATGCGACGACAA	371	(JanssenDuijghuijsen et al., 2017)
β-actin	F: ATGTGGCCGAGGACTTTGATT R: AGTGGGGTGGCTTTTAGGATG	107	(JanssenDuijghuijsen et al., 2017)

#### Statistical analysis

The results are expressed as mean ± SD (standard deviation) of three independent experiments. Statistical differences were determined using one-way ANOVA, performed by GraphPad Prism 5 software version 5.04 (GraphPad Software, Inc., San Diego, CA, USA). Statistical differences are indicated by asterisks (*P <0.05, **P <0.01, ***P <0.001, and ****P <0.0001).

## Results

### Characterization of *L. brevis*-derived MVs

**Figure 1:**
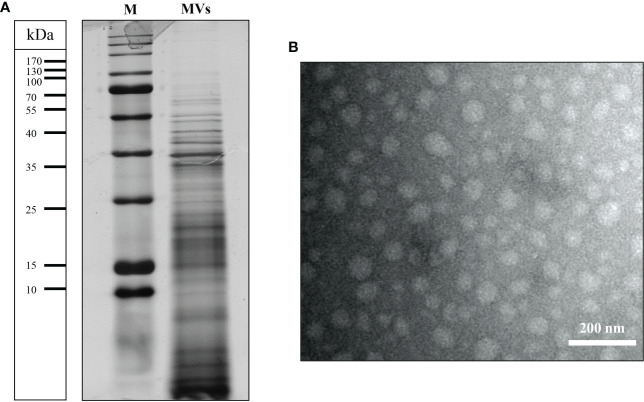
Characterization of the *L. brevis* strain IBRC-M10790-derived MVs isolated in this study. **(A)** Protein profiles of MVs separated by 12% SDS-PAGE followed by visualization of proteins using Coomassie blue staining. Lines to the left indicate the molecular masses of the protein standards in kDa. **(B)** Representative transmission electron micrographs of MVs. Scale bar (lower right) represent: 200 nm. M, molecular weight marker.

*L. brevis*-derived MVs were isolated from the culture supernatant by filtration and ultracentrifugation. The protein profile of *L. brevis*-derived MVs was observed by SDS-PAGE ranging from 10-100 kDa (**Figure 1A**). The isolated MVs were morphologically characterized by TEM analysis, revealing nanosized, spherical, membrane-bound vesicles (**Figure 1B**).

### Cell viability

To evaluate the toxicity of vitamin D3, *H. pylori*, *L. brevis*, and *L. brevis* derivatives, the MTT assay was carried out measuring the viability of AGS cells treated with *H. pylori* (MOI 100), vitamin D3 (20, 50, 100, 150 nmol), live *L. brevis* (MOI 10, 50, and 100), pasteurized *L. brevis* (10^9^ CFU/ml), *L. brevis*-derived MVs (1, 10, 50, and 100 μg/ml), and *L. brevis* CFS (20% (v/v) compared to the untreated control. As demonstrated in **Figure S1**, the viability of AGS cells slightly reduced following *H. pylori* infection and increased after probiotic or vitamin D3 treatment; however, there was no significant alteration in the viability of AGS cells in either group compared to the control. Therefore, considering the results from previous studies (Hu et al., 2019; Zhou et al., 2020), *L. brevis* at MOI 100, *L. brevis*-derived MVs at concentration of 100 μg/ml, and 150 nmol of vitamin D3 were used as the selected concentrations for further cell culture experiments.

### The impact of vitamin D3 and *L. brevis* on *H. pylori* adhesion

**Figure 2:**
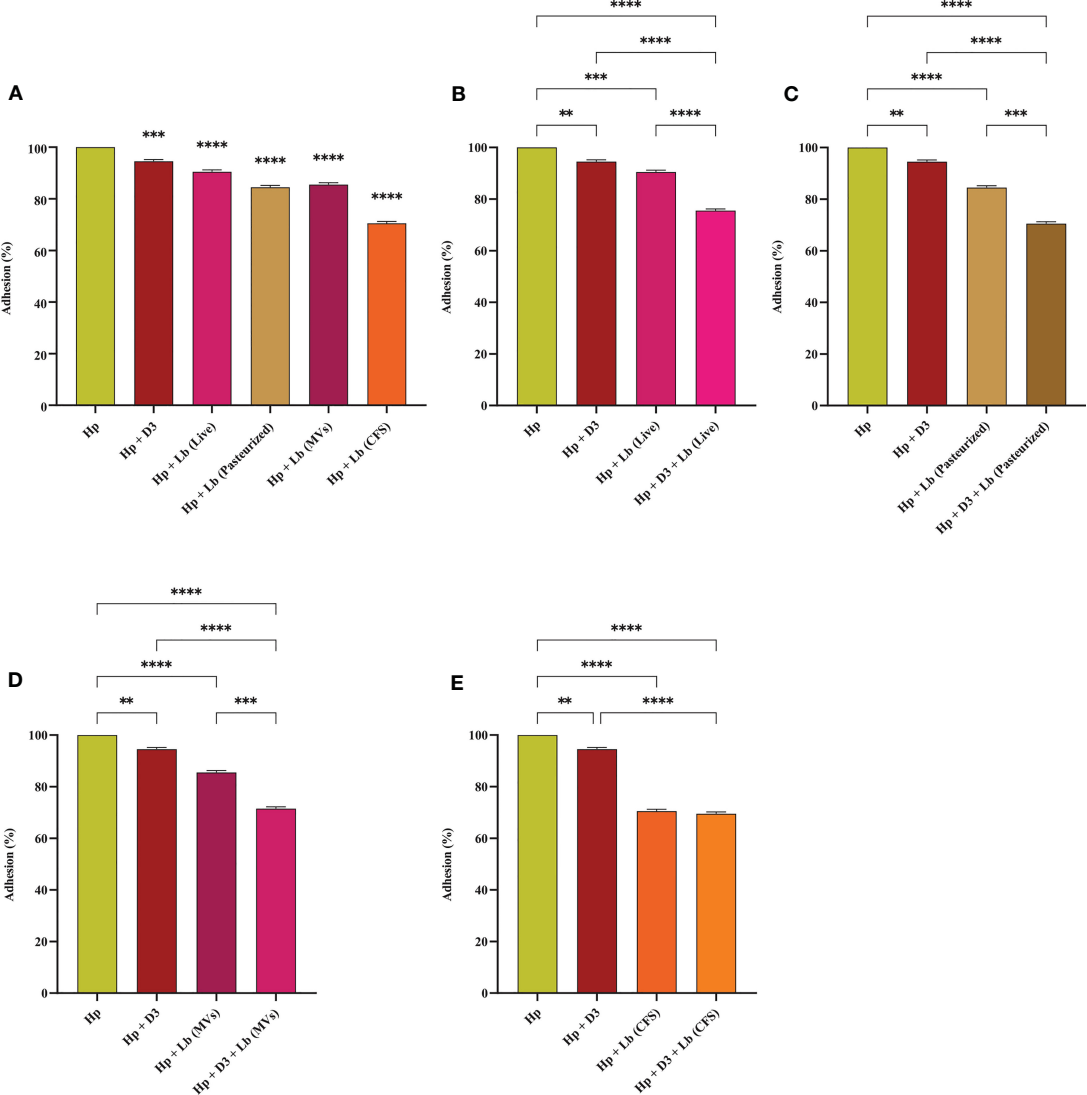
Adherence rate of *H. pylori* to AGS cells. **(A)** Adherence rate of *H. pylori* treated with vitamin D3, live and pasteurized *L. brevis*, *L. brevis*-derived MVs, and *L. brevis* CFS. Adherence rate of *H. pylori* treated with vitamin D3 and live *L. brevis* **(B)**, pasteurized *L. brevis* **(C)**, *L. brevis*-derived MVs **(D)**, or *L. brevis* CFS **(E)**. (*P <0.05; **P <0.01; ***P <0.001; ****P <0.0001).

*H. pylori* adhesion to AGS cells was screened following 2 h of exposure to different vitamin D3 and probiotic treatment groups. Live and pasteurized *L. brevis*, *L. brevis*-derived MVs, and *L. brevis* CFS significantly reduced *H. pylori* adhesion (**Figure 2**). Vitamin D3 treatment slightly decreased *H. pylori* adhesion by approximately 5.5% and was considered statistically significant, which might not be substantial in animal models and clinical trials. The combination of vitamin D3 with live *L. brevis*, pasteurized *L. brevis*, or *L. brevis*-derived MVs significantly reduced *H. pylori* adhesion to AGS cells compared to the same probiotic treatment groups without vitamin D3. The anti-adhesion activity of *L. brevis* CFS, however, presented no significant alteration in combination with vitamin D3. All complementary groups exhibited significantly higher anti-adhesion activity compared to the vitamin D3-treated group. Overall, the adhesion of *H. pylori* to AGS cells was significantly interfered by vitamin D3 or probiotic treatment, especially *L. brevis* CFS. Given the high efficacy of *L. brevis* CFS, vitamin D3 and *L. brevis* CFS combination presented no synergistic effect in preventing *H. pylori* adhesion. However, the combination of vitamin D3 with other *L. brevis* derivatives demonstrated a more significant reduction (up to 30%) in *H. pylori* adhesion.

### The effect of vitamin D3 and *L. brevis* on *H. pylori*-mediated oxidative stress

**Figure 3:**
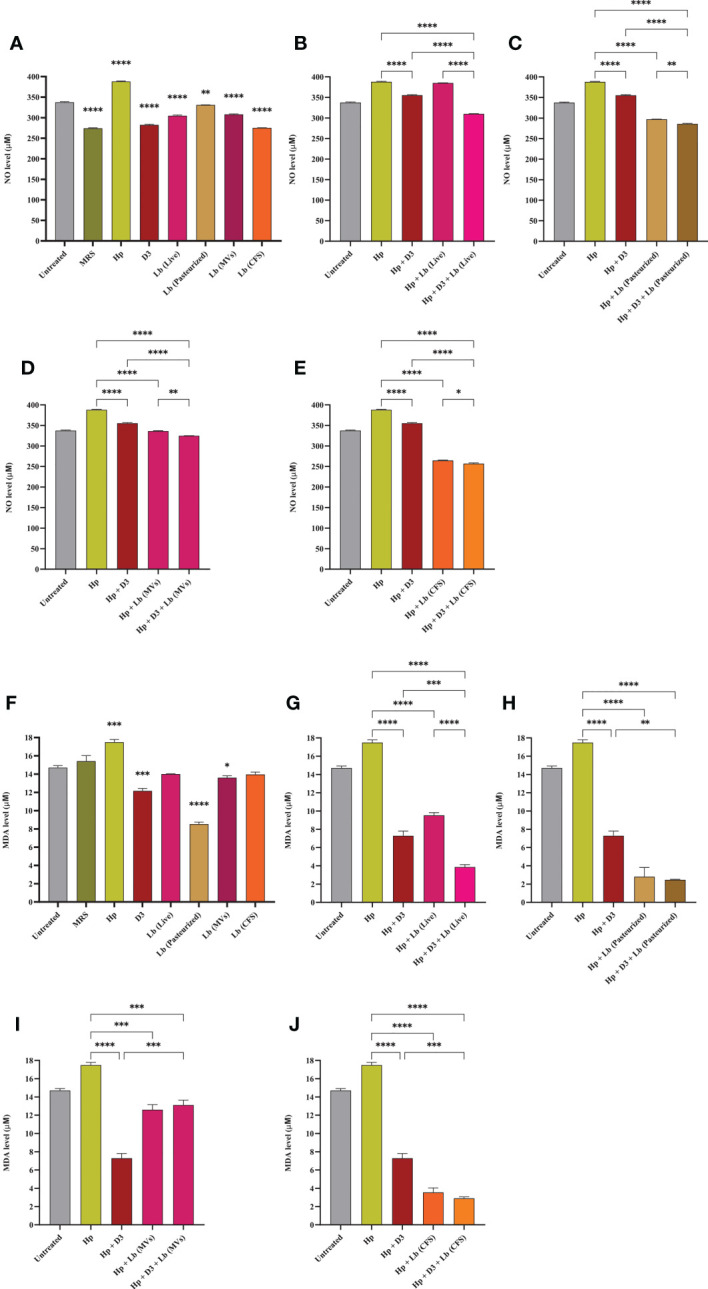
NO and MDA concentration in the culture supernatant of AGS cells. **(A)** NO concentration in the presence of *H. pylori*, vitamin D3, live and pasteurized *L. brevis*, *L. brevis*-derived MVs, and *L. brevis* CFS. NO concentration in the culture supernatant of AGS cells during exposure to *H. pylori* and simultaneous treatment with vitamin D3 and live *L. brevis* **(B)**, pasteurized *L. brevis* **(C)**, *L. brevis*-derived MVs **(D)**, or *L. brevis* CFS **(E)**. **(F)** MDA concentration in the presence of *H. pylori*, vitamin D3, live and pasteurized *L. brevis*, *L. brevis*-derived MVs, and *L. brevis* CFS. MDA concentration in the culture supernatant of AGS cells during exposure to *H. pylori* and simultaneous treatment with vitamin D3 and live *L. brevis* **(G)**, pasteurized *L. brevis* **(H)**, *L. brevis*-derived MVs **(I)**, or *L. brevis* CFS **(J)**. (*P <0.05; **P <0.01; ***P <0.001; ****P <0.0001).

We measured NO and MDA levels in AGS cells to evaluate the antioxidant capacity of vitamin D3 and *L. brevis*. As depicted in **Figure 3**, *H. pylori* infection significantly increased NO concentration in AGS cells, whereas vitamin D3 and probiotic treatment substantially lowered NO levels, compared to *H. pylori*-infected cells. Notably, the combination of vitamin D3 with each type of probiotic treatment group (especially live *L. brevis*) presented a significant reduction in oxidative stress, compared to vitamin D3 or probiotic treatment alone. Likewise, the infection of AGS cells with *H. pylori* resulted in significantly higher levels of MDA, compared to the untreated control (**Figure 3**). Vitamin D3 or probiotic treatment, however, substantially reduced MDA levels, compared to the *H. pylori*-infected group. Compared to vitamin D3 treatment, the combination of vitamin D3 with live *L. brevis*, pasteurized *L. brevis*, and *L. brevis* CFS demonstrated a synergistic effect in lowering MDA concentration. Additionally, live *L. brevis* and vitamin D3 combination exhibited a synergistic effect, compared to probiotic treatment alone. Hence, vitamin D3 and probiotic treatment together (particularly live *L. brevis*) might present a more significant impact on reducing *H. pylori*-induced oxidative stress.

### Vitamin D3 and *L. brevis* promote the integrity of gastric epithelial barrier

**Figure 4:**
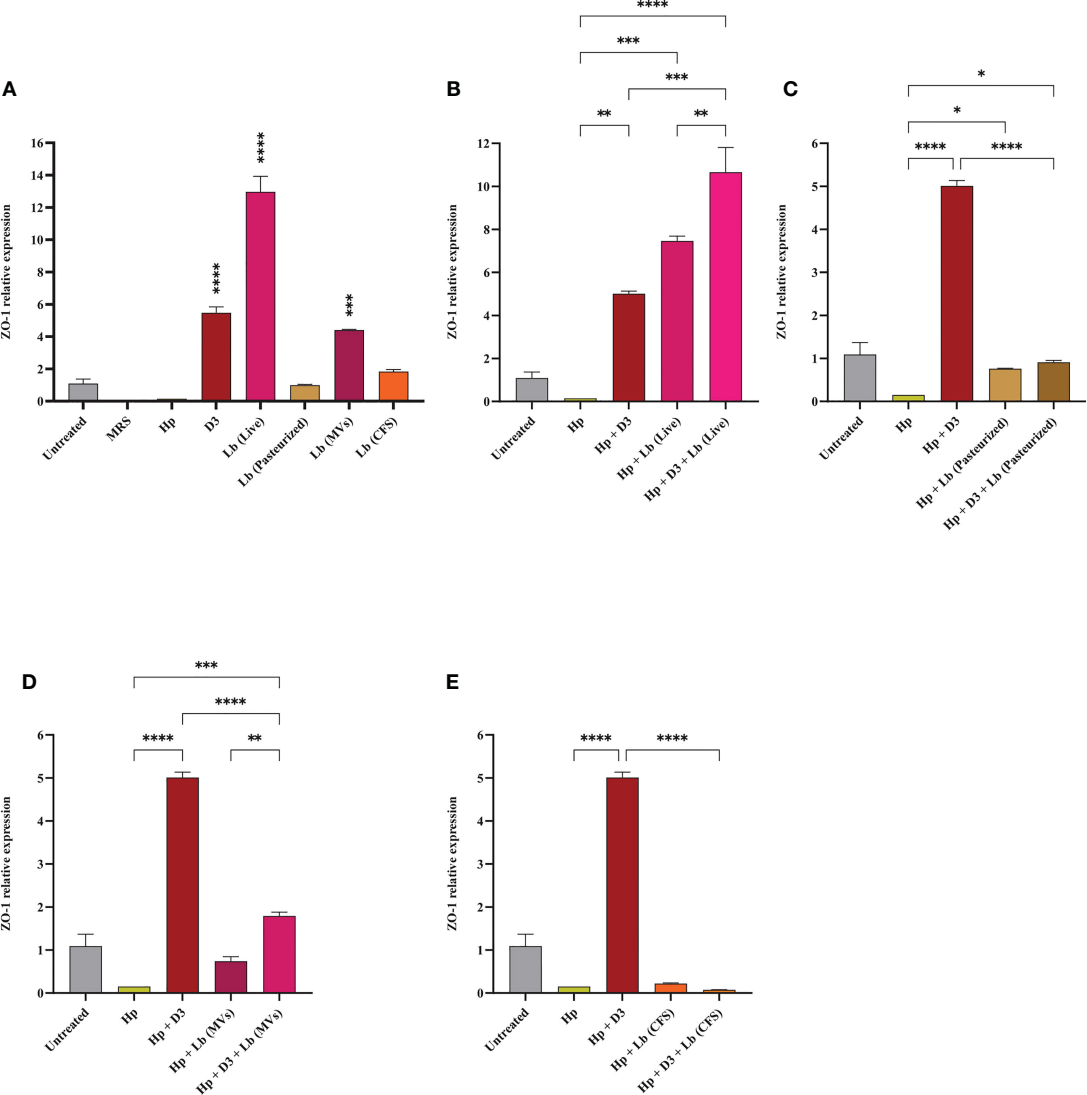
The expression level of ZO-1 in AGS cells. **(A)** ZO-1 expression level in AGS cells treated with *H. pylori*, vitamin D3, live and pasteurized *L. brevis*, *L. brevis*-derived MVs, and *L. brevis* CFS. ZO-1 expression level in AGS cells during exposure to *H. pylori* and simultaneous treatment with vitamin D3 and live *L. brevis* **(B)**, pasteurized *L. brevis* **(C)**, *L. brevis*-derived MVs **(D)**, or *L. brevis* CFS **(E)**. (*P <0.05; **P <0.01; ***P <0.001; ****P <0.0001).

We assessed the expression level of ZO-1 in AGS cells upon *H. pylori* infection and vitamin D3 and/or probiotic treatment. As presented in **Figure 4**, *H. pylori* infection of AGS cells was associated with a substantial reduction in the expression of ZO-1. On the contrary, vitamin D3, live *L. brevis*, and *L. brevis*-derived MVs significantly elevated the expression level of ZO-1 compared to the untreated control. Pasteurized *L. brevis* and *L. brevis* CFS, however, demonstrated no significant alteration in expression of this gene compared to the untreated control. The treatment of *H. pylori*-infected cells with vitamin D3, live *L. brevis*, pasteurized *L. brevis*, and *L. brevis*-derived MVs substantially promoted the expression of ZO-1 compared to the *H. pylori* control group. Furthermore, combination of live *L. brevis* and *L. brevis*-derived MVs with vitamin D3 exhibited a more significant elevation compared to the same probiotic treatment groups. Taken together, live, and pasteurized *L. brevis*, *L. brevis*-derived MVs, and vitamin D3 compensate the reduction of ZO-1 expression upon *H. pylori* infection. The combination of vitamin D3 with live *L. brevis* and *L. brevis*-derived MVs might exert a synergistic effect on the expression of ZO-1 in AGS cells.

### Vitamin D3 downregulates *H. pylori*-induced expression of inflammatory genes

**Figure 5:**
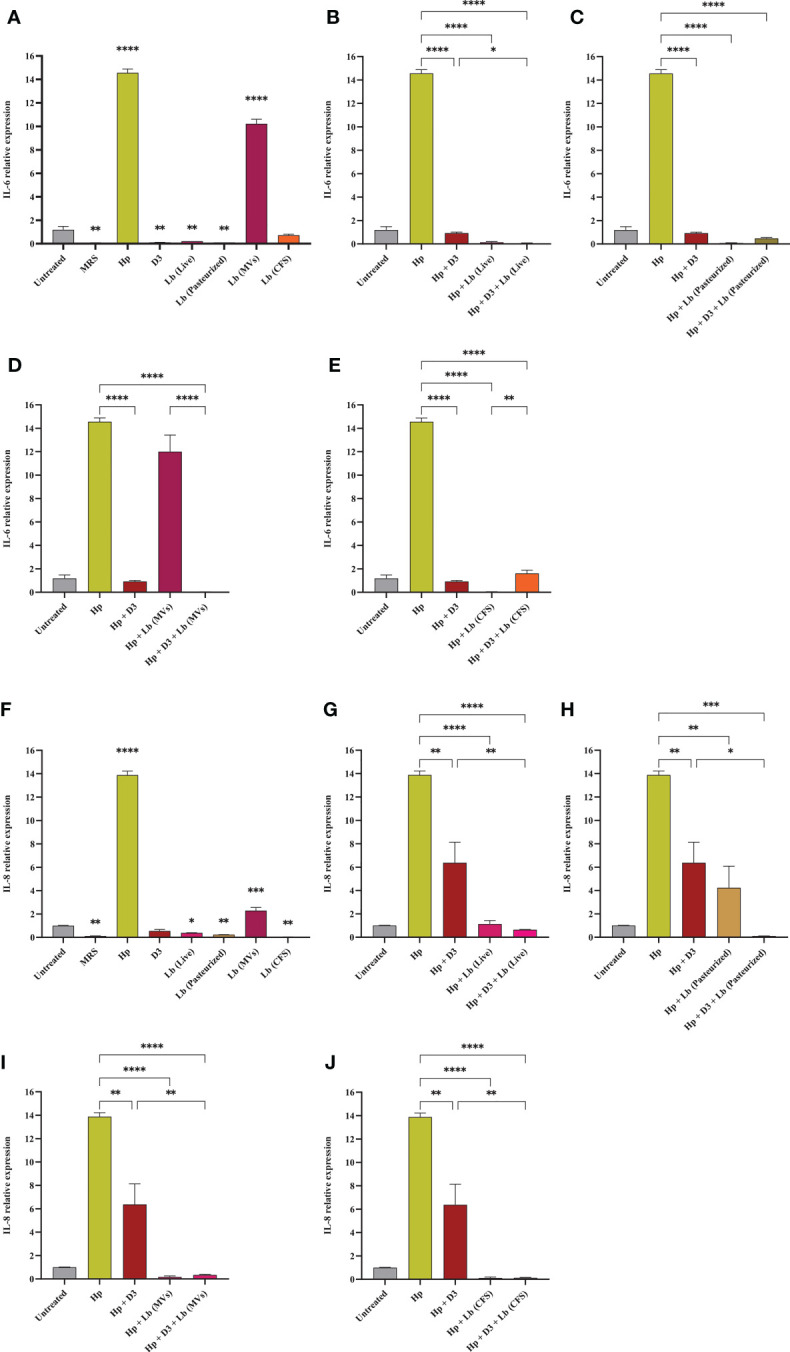
The expression level of IL-6 and IL-8 in AGS cells. **(A)** IL-6 expression level in AGS cells treated with *H. pylori*, vitamin D3, live and pasteurized *L. brevis*, *L. brevis*-derived MVs, and *L. brevis* CFS. IL-6 expression level in AGS cells during exposure to *H. pylori* and simultaneous treatment with vitamin D3 and live *L. brevis* **(B)**, pasteurized *L. brevis* **(C)**, *L. brevis*-derived MVs **(D)**, or *L. brevis* CFS **(E)**. **(F)** IL-8 expression level in AGS cells treated with *H. pylori*, vitamin D3, live and pasteurized *L. brevis*, *L. brevis*-derived MVs, and *L. brevis* CFS. IL-8 expression level in AGS cells during exposure to *H. pylori* and simultaneous treatment with vitamin D3 and live *L. brevis* **(G)**, pasteurized *L. brevis* **(H)**, *L. brevis*-derived MVs **(I)**, or *L. brevis* CFS **(J)**. (*P <0.05; **P <0.01; ***P <0.001; ****P <0.0001).

**Figure 6:**
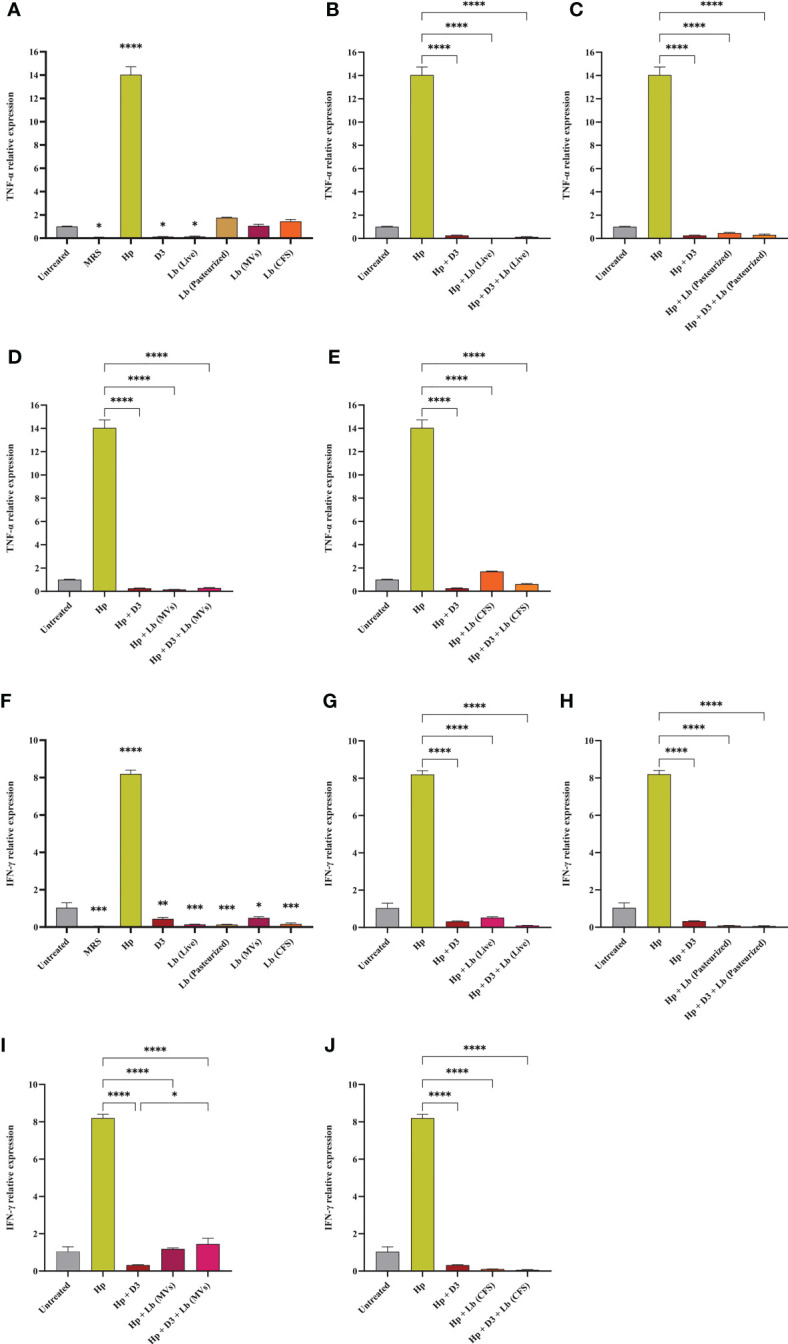
The expression level of TNF-α and IFN-γ in AGS cells. **(A)** TNF-α expression level in AGS cells treated with *H. pylori*, vitamin D3, live and pasteurized *L. brevis*, *L. brevis*-derived MVs, and *L. brevis* CFS. TNF-α expression level in AGS cells during exposure to *H. pylori* and simultaneous treatment with vitamin D3 and live *L. brevis* **(B)**, pasteurized *L. brevis* **(C)**, *L. brevis*-derived MVs **(D)**, or *L. brevis* CFS **(E)**. **(F)** IFN-γ expression level in AGS cells treated with *H. pylori*, vitamin D3, live and pasteurized *L. brevis*, *L. brevis*-derived MVs, and *L. brevis* CFS. IFN-γ expression level in AGS cells during exposure to *H. pylori* and simultaneous treatment with vitamin D3 and live *L. brevis* **(G)**, pasteurized *L. brevis* **(H)**, *L. brevis*-derived MVs **(I)**, or *L. brevis* CFS **(J)**. (*P <0.05; **P <0.01; ***P <0.001; ****P <0.0001).

In this study, we evaluated the potential capacity of vitamin D3 in modulating the expression of pro-inflammatory cytokines IL-6, IL-8, TNF-α, and IFN-γ in AGS cells during *H. pylori* infection (**Figures 5**, **6**). The co-culture of AGS cells with the clinical isolate of *H. pylori* significantly promoted the expression of pro-inflammatory cytokines compared to the untreated control. Treatment of AGS cells with vitamin D3 resulted in a substantial reduction in the expression of TNF-α and IFN-γ compared to the untreated control. Furthermore, treatment of *H. pylori*-infected cells with 150 nmol of vitamin D3 for 24 h substantially lowered the expression of pro-inflammatory cytokines IL-6, IL-8, TNF-α, and IFN-γ.

### *L. brevis* suppresses *H. pylori*-induced expression of inflammatory genes

Here, we investigated the capacity of the local strain *L. brevis* IBRC-M10790 to reduce the inflammatory activity of *H. pylori*. In this regard, live *L. brevis*, pasteurized *L. brevis*, and *L. brevis* CFS significantly lowered the expression level of IL-8 and IFN-γ in uninfected AGS cells compared to the untreated control (**Figures 5**, **6**). However, only live *L. brevis* could substantially reduce the expression of pro-inflammatory cytokine TNF-α (**Figure 6**). On the contrary, *L. brevis*-derived MVs presented a significant overexpression of IL-6 and IL-8 in uninfected AGS cells (**Figure 5**). Live and pasteurized *L. brevis* profoundly, but not statistically significantly, decreased the IL-6 expression (**Figure 5**). Yet, the exposure of *H. pylori*-infected AGS cells to either probiotic treatment groups led to a substantial reduction in the expression of IL-6, IL-8, TNF-α, and IFN-γ.

### The synergistic effect of vitamin D3 and *L. brevis* on *H. pylori*-induced inflammation

Vitamin D3 combination with *L. brevis* CFS significantly promoted the anti-inflammatory activity of this probiotic treatment group in reducing the expression level of IL-6, IL8, and IFN-γ. Evaluating the expression level of IFN-γ, vitamin D3 could substantially boost the anti-inflammatory effect of live *L. brevis* in *H. pylori*-infected AGS cells (**Figure 6G**). Similarly, the combination of vitamin D3 and *L. brevis*-derived MVs exhibited a more significant reduction in the expression of IL-6 in *H. pylori*-infected AGS cells compared to the same probiotic treatment group (**Figure 5D**). On the other hand, each probiotic treatment groups significantly promoted the suppressive activity of vitamin D3 against the expression of IL-6 in *H. pylori*-infected AGS cells (**Figure 5**). Consequently, the combination of vitamin D3 and *L. brevis* to some extent presented a synergistic anti-inflammatory activity during *H. pylori* infection.

## Discussion

**Figure 7:**
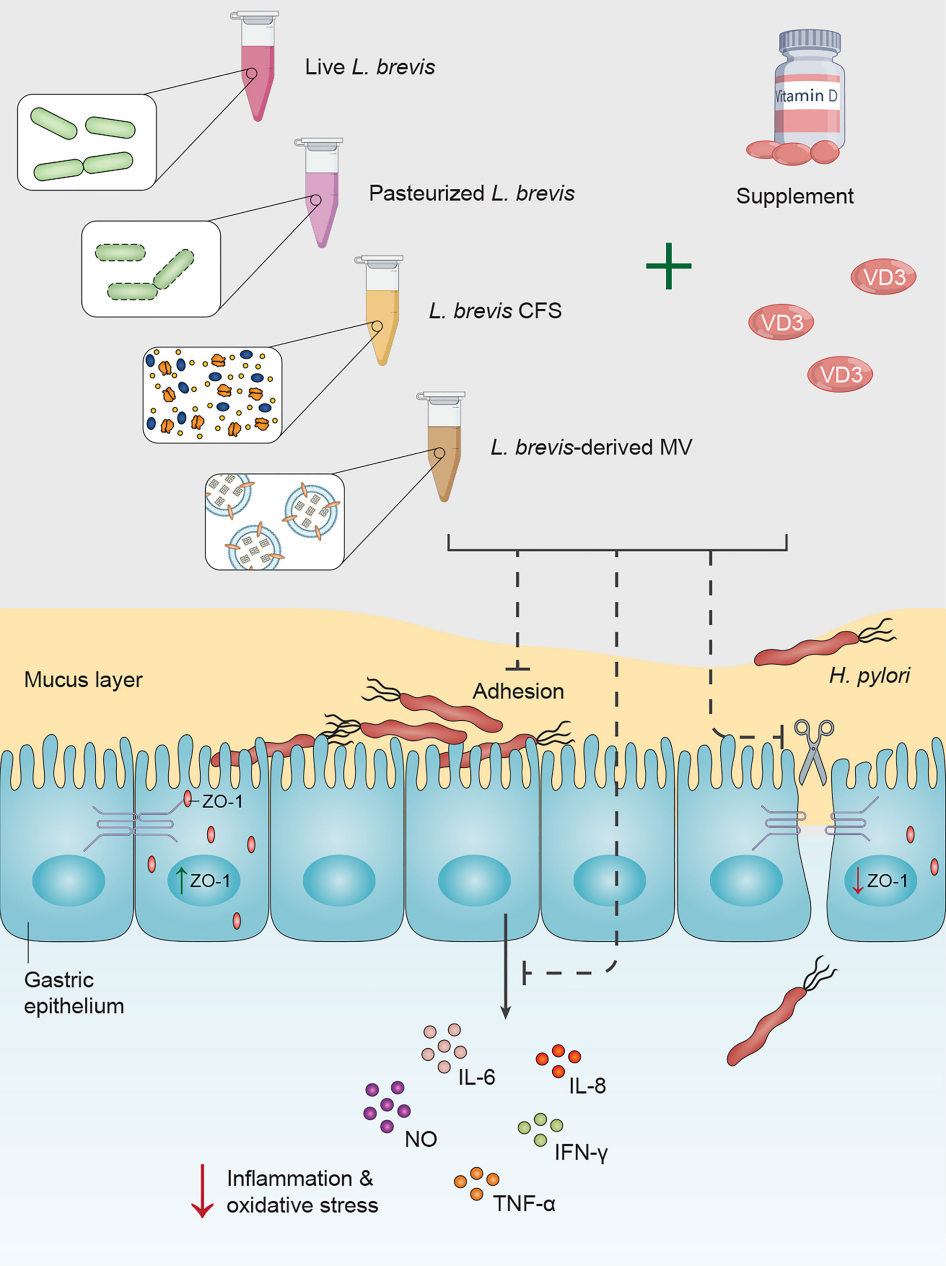
Vitamin D3 and *L. brevis* co-treatment can prevent *H. pylori* attachment to the gastric epithelium, attenuate *H. pylori*-induce inflammation and oxidative stress, and promote the integrity of the gastric epithelial barrier.

The increasing incidence of antibiotic resistance in *H. pylori* is a global threat, leading to a significant reduction in sufficient efficacy of *H. pylori* eradication (>80-90% efficacy level) and an elevation in the risk of clinical complications. All recommended regimens in treatment guidelines as first-line and rescue therapies encounter failure in 10-30% of *H. pylori*-infected subjects (Tshibangu-Kabamba and Yamaoka, 2021). In the context of antibiotic resistance, alternative and complementary therapeutic strategies are being given considerable attention. Probiotic supplementation has demonstrated several beneficial effects by eliminating pathogenic bacteria, promoting host immunity, and attenuating antibiotic side effects (Nabavi-Rad et al., 2022b). Given the beneficial yet insufficient influence of probiotic monotherapy on *H. pylori* eradication, probiotics are taken alongside antibiotics (Losurdo et al., 2018). In an effort to promote probiotic efficacy, recent studies exhibited a synergistic effect of combining probiotics with minerals and vitamins, especially vitamin D (Abboud et al., 2020). Jamilian et al. reported the beneficial influence of probiotics (*Lactobacillus acidophilus*, *Bifidobacterium lactis*, *Bifidobacterium bifidum*, and *Bifidobacterium longum*) and selenium co-supplementation on the general score and insulin, homeostasis model of assessment-insulin resistance (HOMA-IR), quantitative insulin sensitivity check index (QUICKI), fasting plasma glucose (FPG), high-sensitivity C-reactive protein (hs-CRP), total antioxidant capacity (TAC), and glutathione (GSH) serum levels in chronic schizophrenia (Jamilian and Ghaderi, 2021). Additionally, Ghaderi et al. demonstrated the favorable influence of probiotics (*L. acidophilus*, *B. bifidum*, *Lactobacillus reuteri*, and *Lactobacillus fermentum*) and vitamin D administration on the serum MDA levels, triglycerides levels, and total cholesterol/HDL (high-density lipoprotein)-cholesterol ratio in schizophrenia patients (Ghaderi et al., 2019). Raygan and colleagues presented the improvement of mental health and insulin sensitivity of type 2 diabetic patients following 12 weeks of probiotics (*L. acidophilus*, *L. reuteri*, *L. fermentum*, and *B. bifidum*) and selenium co-supplementation (Raygan et al., 2019). Furthermore, the combination of probiotics (*L. acidophilus*, *B. bifidum*, and *Bifidobacterium animalis*) with vitamin C exhibited a beneficial impact on the prevention and treatment of the upper respiratory tract infection (Garaiova et al., 2015). Vitamin D and probiotics (*L. acidophilus*, *B. bifidum*, *L. reuteri*, and *L. fermentum*) co-supplementation was also reported to reduce serum levels of inflammation and oxidative stress biomarkers (hs-CRP and MDA) in gestational diabetes patients (Jamilian et al., 2019). Vitamin D supplementation has been suggested with the capacity to attenuate the serum levels of several inflammatory biomarkers by interacting with the vitamin D receptor (VDR) (Alvarez et al., 2013; Gregório et al., 2021). Vitamin D-VDR interaction was reported to further induce the expression of cathelicidin antimicrobial peptide (CAMP) and inhibit *H. pylori* infection (Zhou et al., 2020). Furthermore, vitamin D activates the autolysosomal degradation of *H. pylori* by interacting with the protein disulfide isomerase family A member 3 (PDIA3) receptor (Hu et al., 2019). Given the negative correlation between *H. pylori* infection and serum levels of vitamin D and the beneficial impact of probiotic supplementation on *H. pylori* eradication, we evaluated the synergistic effect of probiotic and vitamin D treatment on the inflammatory and oxidative activity of *H. pylori*. We further evaluated the influence of probiotic and vitamin D treatment on the integrity of AGS cells and the adherence rate of *H. pylori* to these cells (**Figure 7**). To this end, we utilized live and pasteurized *L. brevis*, *L. brevis*-derived MVs, and *L. brevis* CFS, as well as vitamin D3 in this work.

Upon contacting the host gastric mucus layer, *H. pylori* protects itself by swimming freely through the viscous layer of gastric mucus, attaching to gastric epithelial cells, and colonizing the gastric mucosa epithelium (Huang et al., 2016). Given the significance of *H. pylori* attachment to gastric epithelial cells for its survival, colonization, and pathogenesis, we assessed the effect of *L. brevis* and vitamin D3 treatment on the adherence rate of *H. pylori* to AGS cells. Consistent with previous studies (Chen et al., 2019; Song et al., 2019), *L. brevis* or vitamin D3 treatment substantially reduced *H. pylori* attachment to AGS cells. Furthermore, vitamin D3 co-treatment with live and pasteurized *L. brevis* and *L. brevis*-derived MVs presented a synergistic impact on the adherence rate of *H. pylori*. One potential mechanistic action of live probiotics is competitive exclusion of *H. pylori*, which refers to rigorous competition for attachment sites and available nutrients (van Zyl et al., 2020). As for probiotic derivatives and vitamin D3, they possibly can reduce the number of *H. pylori* bacteria through bactericidal activity and further decrease *H. pylori* adherence rate by regulating the integrity of the gastric epithelial barrier (Raheem et al., 2021; Nabavi-Rad et al., 2022a).

The gastric epithelial barrier, which is a major obstacle preventing *H. pylori* penetration to lamina propria, plays a key role in preserving gastric homeostasis. The integrity of this barrier highly relies on the expression and production of tight junction proteins (Moonwiriyakit et al., 2022). The tight junction protein 1 (TJP1)/ZO-1 has a critical activity in maintaining the integrity of the gastric barrier and repairing the mucus layer (Cario et al., 2004; Kuo et al., 2021). *H. pylori* separates the epithelial tight junction proteins, disrupts the membrane distribution of ZO-1 in gastric epithelial cells, and leads to the accumulation of ZO-1 in small vesicles (Wroblewski and Peek, 2011). Yeung et al. reported a negative correlation between vitamin D deficiency and the production of tight junction proteins in the gut epithelium of mouse models (Yeung et al., 2021). Zhao et al. also demonstrated the protective role of vitamin D3 in reducing mucosal injury and maintaining the structural integrity of colonic epithelial cells in acute colitis mouse models (Zhao et al., 2012). Similarly, our results exhibited the substantial influence of vitamin D3 treatment on the expression level of ZO-1 in AGS cells. Moreover, the combination of vitamin D3 with live *L. brevis* or *L. brevis*-derived MVs had a remarkable impact on *H. pylori*-infected AGS cells, compared to probiotic treatment alone.

Following *H. pylori* attachment to the gastric epithelium and penetration to the lamina propria, the excessive production of reactive oxygen species (ROS), reactive nitrogen species (RNS), and pro-inflammatory cytokines from the epithelium and immune cells have a particular significance in *H. pylori* pathogenesis (Jain et al., 2021). *H. pylori*-induced activation of pro-inflammatory pathways acts mainly through nuclear factor kappa B (NF-κB), which leads to the accumulation of reactive radicals and the development of precancerous lesions (He et al., 2022). Continuous oxidative stress can induce DNA damage, cellular apoptosis, and consequently gastric carcinogenesis (Butcher et al., 2017). Using VDR knockdown mouse models, Wu et al. reported that VDR expression negatively regulates pathogen-induced NF-κB activity and attenuates the inflammation (Wu et al., 2010). Furthermore, different strains of *L. brevis* bacteria have been reported to present a beneficial impact by modulating the immune response and attenuating the intensity of oxidative stress and inflammation (Lim et al., 2016; Jiang et al., 2018; Brandi et al., 2020). Compared to the beneficial effect of live *L. brevis*, the combination of *L. brevis* with vitamin D3 demonstrated a significant reduction in the concentration of oxidative biomarkers. On the other hand, vitamin D3 combination with live *L. brevis*, pasteurized *L. brevis*, or *L. brevis* CFS presented a synergistic effect on *H. pylori*-infected AGS cells, compared to the vitamin D3 treatment group. Although vitamin D is better known for its capacity in eradicating *H. pylori* infection, this supplement has potent anti-inflammatory traits by regulating the expression of different pro- and anti-inflammatory biomarkers (Krajewska et al., 2022). In this study, vitamin D3 treatment resulted in a lower expression level of pro-inflammatory cytokines in *H. pylori*-infected AGS cells with a significant reduction in TNF-α and IFN-γ expression levels. Additionally, vitamin D3 co-treatment with *L. brevis* to some extent could boost the anti-inflammatory properties of this probiotic bacteria.

## Conclusions

This study demonstrates a synergistic effect for vitamin D3 and *L. brevis* co-treatment in reducing *H. pylori* inflammatory and oxidative activity as well as adherence ratio in AGS cells. However, limitations to our work include the absence of *in vivo* experiments and proteomics/metabolomics analysis. The metabolite composition of *L. brevis*-derived MVs, the bactericidal mechanistic action of *L. brevis* against *H. pylori*, and the efficacy of vitamin D3 combination with multi-strain probiotic consortia on *H. pylori* infection need further elucidation. Furthermore, studying the influence of vitamin D3 and *L. brevis* on gastric and fecal bacterial metabolites, especially *H. pylori* metabolites, could bring up important insights about *H. pylori* treatment. Therefore, *in vivo* studies and mechanism-oriented clinical trials are required to investigate the influence of probiotics and vitamin D co-supplementation on different aspects of *H. pylori* infection.

## Data availability statement

The original contributions presented in the study are included in the article/**Supplementary Material**. Further inquiries can be directed to the corresponding author.

## Author contributions

AN-R, SJ, and MA performed the *H. pylori* and probiotic culture, cell culture and molecular assays. AN-R reviewed the literature and wrote the manuscript draft. AY contributed to study design, conceptualization and methodology. AN-R and AY analyzed and interpreted the data. AY, KR, TM, and MZ critically edited the manuscript. All authors contributed to the article and approved the submitted version.
