# Supplementary material for: The synergistic effect of Levilactobacillus brevis IBRC-M10790 and vitamin D3 on Helicobacter pylori-induced inflammation
Source: Front Cell Infect Microbiol. 2023 May 5;13:1171469. doi: 10.3389/fcimb.2023.1171469 (PMC10196258; doi:10.3389/fcimb.2023.1171469)
Supplement: Supplementary file 1 [file DataSheet_1.docx]

**
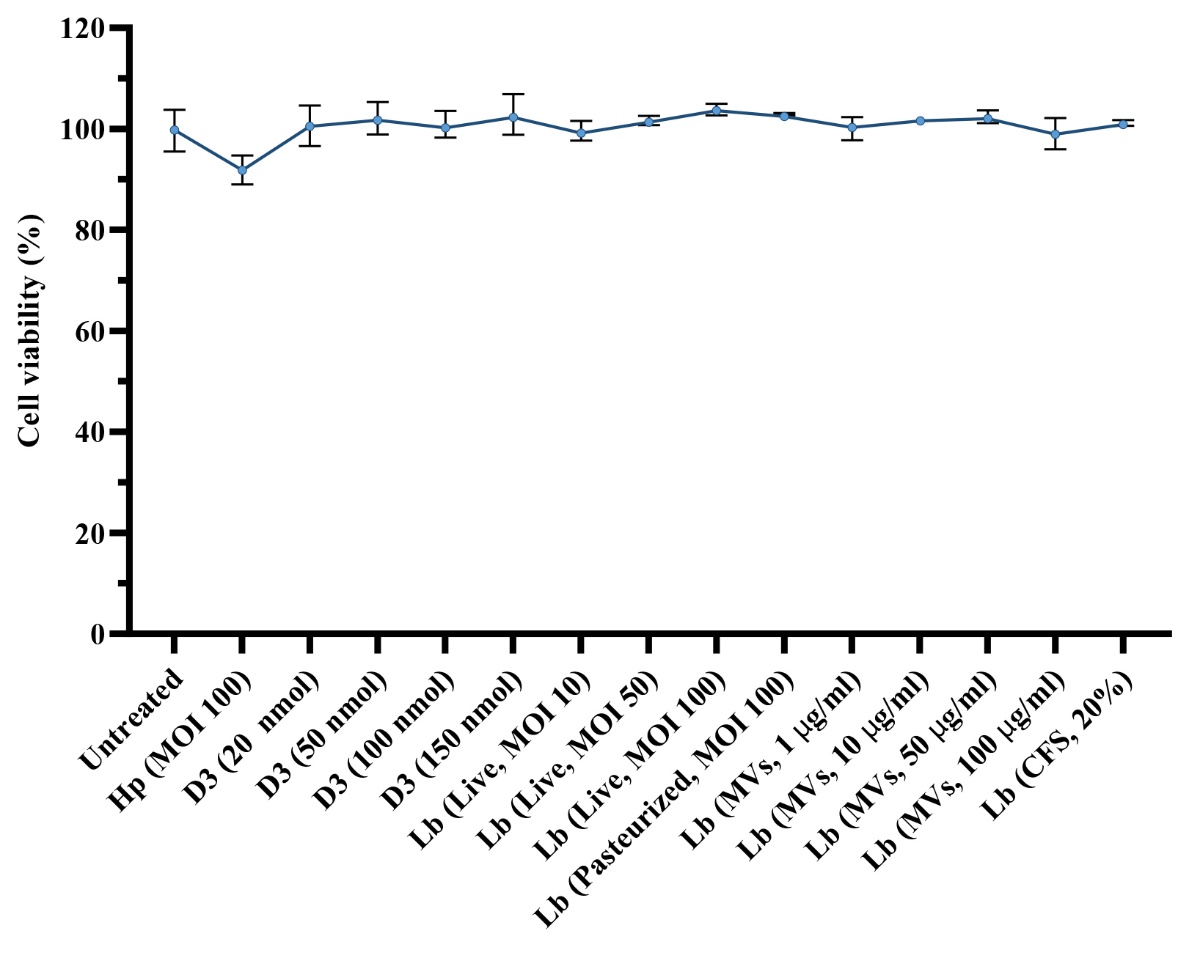
**

**Figure S1** Cell viability was determined by MTT assay for AGS cells treated with *H. pylori* (MOI 100), vitamin D3 (20, 50, 100, 150 nmol), live *L. brevis* (MOI 10, 50, and 100), pasteurized *L. brevis* (10^9^ CFU/ml), *L. brevis*-derived MVs (1, 10, 50, and 100 μg/ml), and *L. brevis* CFS (20% (v/v)) for 24 h. (**P* <0.05; ***P* <0.01; ****P* <0.001; *****P* <0.0001).
